# Mechanism of anticancer action of *bifidobacterium*: Insights from gut microbiota

**DOI:** 10.18632/oncotarget.28779

**Published:** 2025-11-14

**Authors:** Hoang Do, Esther Asiamah, Mayanijesu Olorife, Arathi Pillai, Sakshi Patel, Ponniah Selvakumar, Sidhartha D. Ray, Ashakumary Lakshmikuttyamma

**Affiliations:** ^1^Department of Pharmaceutical Sciences, Jefferson College of Pharmacy, Thomas Jefferson University, Philadelphia, PA 19107, USA; ^2^Department of Pharmaceutical and Biomedical Sciences, Touro College of Pharmacy, NY 10036, USA

**Keywords:** cancer, gut microbiota, *bifidobacterium*

## Abstract

*Bifidobacterium* has captured major attention recently because of its health benefits and extensive research highlighting its potential in cancer treatment and prevention. Evidence suggests that *bifidobacterium* can actively fight against various types of cancer, including those of the colon, lungs, breast, and stomach. Research indicates that several species of *bifidobacterium* can potentiate the action of chemotherapy, immunotherapy and radiation therapy in battling tumors, and reducing their adverse effects. Bifidobacteria shows its multipronged effect by modulating various immunomodulatory and inflammatory signaling pathways, potentially leading to the suppression of tumor growth. Moreover, different species of bifidobacteria are known to regulate signaling molecules involved in promoting apoptosis. In addition, bifidobacteria have an impact on the regulation of diverse microRNAs. The anticancer properties of *bifidobacterium* may also stem from its ability to detoxify carcinogens and transform dietary elements. This review also covers how dietary factors can influence the prevalence of *bifidobacterium* in the gut, further affecting its anticancer capabilities.

## INTRODUCTION

*Bifidobacteria* have been found in various environments, including the gastrointestinal tract of different mammals, the oral cavity, and the guts of insects [1, 2]. They were first isolated in 1899 from the feces of breast-fed infants [3]. *Bifidobacterium* species, promoted by breastfeeding, produces aromatic lactic acids in infants’ guts which is believed to support immune function early in life. A reduction in specific microbes, such as *Bifidobacterium*, during early life has been associated with a higher risk of developing allergies and asthma in childhood. It has been suggested that depletion of this specific bacterium weaken immune function and increase susceptibility to infectious diseases. [4–6].

*Bifidobacterium* plays a crucial role in human health by modulating myriads of biochemical and physiological networks within the human body, of which few are known but many remain unknown [7]. Various biological functions of *bifidobacteria* have been documented, including immune regulation, anti-tumor activity, anti-pathogenic action, anti-inflammation, anti-aging effects, and regulation of hyperlipemia. [7–10]. Dietary polysaccharides or oligosaccharides, primarily the indigestible components, serve as the energy source for *bifidobacterium* growth [7, 11–12]. Dietary sources profoundly influence *bifidobacteria*. Some reports suggest that supplementation with pectic oligosaccharides (POS) increases specific populations of gut microbiota *in vivo*. Bindels et al. found that POS significantly increases *Bifidobacterium spp., Roseburia spp*., and *Bacteroides spp*. [13]. Another study suggested that all rye-supplemented diets enhanced the *in vivo* growth of *bifidobacterium* compared to non-fiber diet [14]. This study also observed higher levels of plasma enterolactone in the rye-bran group compared to the non-fiber diet group.

## ANTICANCER ACTION OF *BIFIDOBACTERIUM*

Several provocative studies have shown *bifidobacteria’s* potential to act as an effective anticancer agent, in addition to reducing intestinal and liver disorders, boosting immune responses, and deescalating the rapidity of aging [7, 9, 15–20]. Due to their presence in various organs besides the intestine, their anticancer benefits have been studied in different tissues. The enrichment of *Bifidobacterium breve* (*B. breve*) has been reported to inhibit the occurrence and progression of various cancers. [21, 22]. A large volume of literature vouches for the anti-cancer action of *bifidobacterium*.

### *Bifidobacterium* and lung cancer

Majority of lung cancer patients, approximately 90%, belong to non-small cell lung cancer (NSCLC) group. Healthy subjects and lung cancer patients display differences in their gut microbiota. An enhancement of *Bifidobacterium. breve* has been identified in healthy subjects compared to those with non-small cell lung cancer (NSCLC) patients [23]. Further studies identified that median progression-free survival was prolonged in non-small-cell lung cancer (NSCLC) patients with significant levels of *Bifidobacterium. breve* in their gut microbiota [24]. Consistent with these findings, additional studies demonstrated that treatment with aqueous extracts of various *bifidobacterium* species significantly inhibited cell proliferation and induced apoptosis in different NSCLC cell lines (A549, H1299, and HCC827) [25]. These authors suggested that mechanistically *Bifidobacterium bifidum (B. bifidum)* treatment reduced cancer cell invasion by downregulating MMP-9 expression. Therefore, it appears likely that use of bifidobacterium could be developed as an adjunctive anticancer treatment option [25]. Moreover, gut microbiota may likely predict the efficacy and adverse effects of immune checkpoint inhibitor therapy. The presence of *Bifidobacterium, Escherichia*, and *Sarterella* is associated with higher clinical benefits when anti-PD1 immunotherapy is combined with chemotherapy. Interestingly, presence of *Bifidobacterium. breve* in the gut microbiota extended the median progression-free survival of patients treated with anti-PD-1 therapy combined with chemotherapy [24]. NSCLC patients with higher levels of *Parabacteroides, Clostridia bacterium UC5.1_2F7*, and *Bifidobacterium dentium* (*B*. *dentium)* showed a better outcome to checkpoint inhibitor therapy [26]. Another study found that *Bifidobacterium. bifidum* was also abundant in non-small-cell lung cancer patients responsive to checkpoint inhibitor therapy. *Bifidobacterium. bifidum* treatment synergistically induced the anti-cancer action of PD-1 antibody or oxaliplatin therapy in a mouse lung cancer model [27]. Yang et. al. showed increased radiosensitivity of lung cancer when the NSCLC patients were treated with *Bifidobacterium infantis (B. infantis)* [28]. Further clinical studies are warranted to assess the beneficial effect of *bifidobacterium* with other therapies on the reduction of tumor growth and metastasis.

### *Bifidobacterium* and breast cancer

Breast cancer is one of the most common malignancies affecting women worldwide. Approximately 80% of early-detected, non-metastatic breast cancers are curable. Assessing ER (Estrogen Receptor), PR (Progesterone Receptor), and HER2 (Human Epidermal Growth Factor Receptor 2) are the hallmarks for determining whether a patient is eligible for hormonal or anti-HER2-targeted therapies. The health benefits of probiotics, including *bifidobacterium*, have been investigated in breast cancer patients (Stage I–III). When these patients received *Bifidobacterium. longum (B. longum)*, *Lactobacillus acidophilus*, and *Enterococcus faecalis* in combination with docetaxel-based chemotherapy, considerable reduction in plasma LDL and body weight was observed in probiotics treated patients compared to control group [29]. Another study demonstrated that administering probiotics (*Bifidobacterium. longum BB536, Lactobacillus rhamnosus HN001*) along with a mediterranean diet led to reduction in body weight, glucose levels, and insulin resistance in breast cancer survivors [30]. Furthermore, a study using breast cancer survivors found that the combination of *bifidobacterium* and perilla oil decreased the fear of cancer recurrence [31]. Another study showed that combination of *Bifidobacterium. longum* and ononis hirta methanol-extract treatment was effective in enhancing the immune response with a simultaneous reduction in mammary gland tumors in a mouse model [32].

Dynamic changes of gut microbiota are common in premenopausal and postmenopausal women. A recent study identified that *bifidobacterium spp*. was specifically decreased in premenopausal breast cancer patients compared to postmenopausal breast cancer patients [33]. Triple negative breast cancer (TNBC) is a subtype where ER, PR, and HER2 are negative. Hormonal and anti-Her2 therapies remain ineffective for TNBC patients. Epidemiological data indicate that TNBC occurs most often in young women in the premenopausal stage [34]. Young TNBC patients frequently exhibit BRCA1 and BRCA2 mutations. Coincidentally, TP53 and RAD50 mutations are also associated with TNBC in young patients [33]. Studies on the impact of gut microbiota in premenopausal breast cancer are limited. *Bifidobacterium. infantis* 35624 reduced tumor growth in TNBC animal model. Furthermore, *Bifidobacterium. infantis* 35624 and doxorubicin co-administration enhanced the anticancer potential of doxorubicin [35]. Another study using a BALB/c mouse model also found that sonication-killed *Bifidobacterium. bifidum* reduced tumor incidence and progression of TNBC. This bacterium also increased p53 levels and, at the same time, reduced Ki-67 expression [36]. Another study reported the physiological properties of gut bacterial membrane vesicles (B-MVs) and their combined action with cancer immunotherapy in a TNBC mouse model [37]. In a mouse study, *bifidobacterium*-derived B-MVs inhibited the growth of MDA-MB-231-induced triple-negative breast tumors. The antitumor effect of B-MVs is achieved by inducing cancer cell apoptosis through the upregulation of Bax and downregulation of Bcl-2 [37]. Treatment with *Bifidobacterium. longum*-C-CPE-PE23 (a genetically modified strain of *B. longum* secreting claudin-4 (CL-4) targeting protein C-CPE-PE23) to a TNBC mouse exhibited higher induction of apoptotic cells and tumor growth inhibition compared to control groups. Systemic toxicity was minimal in experimental animals with *Bifidobacterium. longum*-C-CPE-PE23 treatment [38].

Currently, options for TNBC treatment are extremely limited, and more carefully designed studies are needed to explore the action of *bifidobacterium* in enhancing the anticancer action of chemotherapy, radiation therapy and immunotherapy.

### *Bifidobacterium* and colorectal cancer

Colorectal cancer (CRC) is the third leading cause of cancer-associated death in men and fourth cause in women. There is ample evidence available on the correlation between CRC and gut microbiota [21, 39]. The ameliorative role of probiotics, including *lactobacillus* and *Bifidobacterium spp*. on CRC has been reported both *in vitro* and *in vivo* [40, 41]. One study investigated the presence of a specific fecal bacteria in colorectal cancer patients and healthy subjects. *Bifidobacterium adolescentis* (*B. adolescentis)* was found to be significantly lower in CRC patients compared to healthy controls [42]. This suggests the benefit of this bacterial population against CRC. The apoptosis-inducing ability of *bifidobacterium* has been demonstrated in the colon cancer cell line LS174T compared to IEC-18 normal cells, although actual mechanisms remain unclear. One study used individual and mixed probiotics, including (1) single strain of *Lactobacillus. reuteri*, (2) *B. Breve*, (3) mixture of 5 strains of *Lactobacilli* (LC), (4) 5 strains of *bifidobacteria* (BC), (5) 10 strains of *Lactobacillus* and *Bifidobacterium* (L+B). Among these probiotics, a cocktail of 5 strains of *bifidobacterium* showed higher apoptosis in LS174T cells. *Bifidobacterium* decreased the expression levels of EGFR, HER-2, and COX-2. The colon cancer incidence and progression of tumor stage were significantly inhibited by *Bifidobacterium* in an animal study [21, 39]. In order to establish a correlation between gut microbiota and CRC, another study isolated *bifidobacterium* from dairy products, infant feces, and probiotic capsules. The cell-free supernatant of isolated *bifidobacterium* was tested for its cytotoxicity in colon cancer cell lines. *Bifidobacterium. bifidum* inhibited the growth of colon cancer cell line SW742 cell-line in particular [43]. *Bifidobacterium* also exhibited regulation of miRNAs and their target genes in an azoxymethane-induced colon cancer mouse model. Treatment of both *Lactobacillus acidophilus* and *Bifidobacterium. bifidum* increased the miR-26b and miR-18a expressions, which were decreased in azoxymethane-induced colon tumors. Further, both the bacterial populations elevated the expression of miR-155. KRAS is one of the targets for miR-155, and the probiotics treatment significantly increased KRAS expression level in the colon tumor. Regulation of these miRNAs and target genes provided potential tumor suppressive action in the colon cancer cells by these probiotics [44]. In contrast, a study found that *Bifidobacterium. longum* (BL) decreased oncomiRs miR-21a and miR-155 but enhanced the tumor suppressor miRNAs (miR-145 and miR-15a) in colorectal cancer mice model [45]. Induction of tumor suppressing miRNAs and inhibition of oncomirs resulted in decreased CRC cell proliferation and invasion [45]. Increased levels of cholesterol, triacylglycerol, and low-density lipoprotein are associated with colorectal cancer. Administration of *Lactobacillus acidophilus* and *Bifidobacterium. bifidum* significantly reduced these lipid molecules in azoxymethane-induced colon tumors. Furthermore, these probiotics decreased vitamin D receptor (VDR) and leptin receptor (LPR) gene expression levels in colon tumors. Regulation of VDR, LPR, and lipid parameters play a key role in the development of colon cancer [46]. Another study also demonstrated the effect of bifidobacterium in reducing colorectal cancer tumorigenesis. Administration of *Bifidobacterium. bifidum* CGMCC 15068 regulated metabolites involved in different pathways such as glycolysis, citrate cycle, fatty acid biosynthesis, butyrate metabolism, and galactose metabolism in a colorectal cancer mouse model. Polysaccharide fractions comprising chiro-inositol, rhamnose, glucose, galactose, and ribose isolated from *Bifidobacterium. bifidum* BGN4 reduced the proliferation of colon cancer HT-29 and HCT-116 cells [47]. Another *bifidobacterium* species, *Bifidobacterium. adolescentis* SPM0212 reduced the growth of colon cancer cell lines, such as, Caco-2, HT-29, and SW480 [48]. Further studies are warranted to explore *bifidobacterium’s* role in supporting chemotherapy, immunotherapy and radiotherapy.

### Gastric cancer and *bifidobacterium*

Gastric cancer is classified into two subtypes such as diffuse and intestinal. The overall survival rate is better for intestinal gastric cancer subtype compared to diffuse type. A study was carried out to analyze the intestinal microflora closely associated with gastric cancer and found that the gastric cancer patients had significantly lower levels of *faecalibacterium, bifidobacterium*, and *subdoligranulum* species in their intestinal tract compared to healthy individuals [49]. Another study showed that probiotics such as *bifidobacterium, lactobacillus*, and *streptococcus* species reduced the gastric cancer associated inflammation [50]. *Helicobacter pylori* infection is one of the prime causes for the development of gastric cancer. There is evidence suggesting that the presence of gut bifidobacterium protected from *Helicobacter pylori* associated gastric diseases [51]. Nada et al. found that *Lactobacillus acidophilus* and *Bifidobacterium. longum* exhibited antiproliferative and anti-angiogenic action against gastric and bladder cancers [52]. More detailed study is required to unravel the beneficiary action of *bifidobacterium* against gastric cancer.

### *Bifidobacterium* and oral squamous carcinoma

Oral carcinoma can occur in mouth, lips, tongue, and gums. Oral squamous cell carcinoma is a tumor that originates from the oral cavity squamous epithelial cells lining. Very few studies are available associated with *bifidobacterium* and oral squamous carcinoma. Li et al. reported that *Bifidobacterium. Breve, another species of bifidobacterium*, inhibited the proliferation of oral squamous carcinoma tumor transplanted in C3H/HeN mice. The antitumor action of *bifidobacterium* is due to the recruitment of T cells to the tumor microenvironment through the secretion of higher levels of IL-12 from dendritic cells [53]. An *in vitro* study using head and neck squamous cell carcinoma cell line observed that exopolysaccharides composed of rhamnose (Rha), arabinose (Ara), galactose (Gal), glucose (Glc), and mannose (Man) derived from *Bifidobacterium. breve* lw01 induced cell cycle arrest and cell apoptosis [54]. Although all these observations significantly testify a positive role for *bifidobacteria*, carefully designed clinical studies are required to explore the beneficial effect of *bifidobacterium* against oral squamous carcinoma.

## MECHANISM OF ANTI-CANCER ACTION OF *BIFIDOBACTERIUM*

The anticancer action of *bifidobacterium* follows a multitude of pathways and mechanisms such as biotransformation, activation of immune function, fermentation of undigested complex polysaccharides, and regulation of various oncogenic and tumor suppressor molecules.

### Immune modulation by *bifidobacterium*

*Bifidobacterium* exhibits tumor-suppressing effects by enhancing immune function by impacting immune cells such as B lymphocytes, NK cells, and macrophages via a variety of pathways and mechanisms [18, 55, 56]. *Bifidobacterium*. *animalis* F1-7 decreased the proliferation of melanoma B16-F10 cells by inhibiting the pro-inflammatory molecules such as IL-6, IL-8 and TNF-α, and activating anti-inflammatory molecule IL-10 [56]. Butanol extract of *Bifidobacterium. adolescentis* SPM0212 reduced the growth of colon cancer cell lines (Caco-2, HT-29, and SW480) and enhanced the TNF-α release [48]. Another study found that supplementation of *Bifidobacterium. adolescentis* reduced colorectal tumor formation by increasing CD143+ cancer-associated fibroblasts, increased GAS1 expression and Wnt/β-catenin pathway [42].

Most of the cancer cells have higher NF-kB activity, which produce higher amounts of pro-inflammatory factors and induces cancer cell proliferation. A recent study found that lipopolysaccharide (LPS) induced protein expression of TLR4 and NF-kB p65 was decreased by *Bifidobacterium. longum* BL-10 group treatment [57]. Furthermore, this study showed that BL-10 treatment balanced the Th1 and Th2 function and reduced the expression levels of different pro-inflammatory cytokines (IFN-γ, IL-2, IL-6, IL-17, IL-22, and IL-12). Conversely, BL-10 enhanced anti-inflammatory cytokine IL-4 levels, which was decreased by LPS treatment [57]. Consumption of probiotic *Bifidobacterium. breve* BR03 and B632 strains decreased TNF-α levels in children with celiac disease [58]. An *in vitro* study using Caco-2 cells found that *Bifidobacterium. animalis* subsp. lactis strain BB12 reduced the TNF-induced production of IL-8 via transcriptional inhibition of NF-kB pathway [59]. Pro-inflammatory cytokines such as TNFα, IL-6, IL- 1β, IL-18, IL-22, and IL-9 were significantly reduced in the colon homogenates of mice treated with *Bifidobacterium. adolescents* compared to controls, whereas the anti-inflammatory cytokines IL-10, IL-4 and IL-5 were higher [60]. *Bifidobacterium. breve* also has been shown to enhance IL-10-production (Type 1 regulatory cells) in Tr1 cells in the large intestine [61]. One study using porcine intestinal epithelial cells challenged with heat-killed enterotoxigenic *Escherichia coli* displayed that *Bifidobacterium. longum BB536* and *B. breve M-16V* strains significantly reduced interleukin (IL)-8, monocyte chemotactic protein (MCP)-1 and IL-6 expression levels [62]. One of our earlier reviews also discussed the action of *bifidobacterium* in downregulating all proinflammatory cytokines through the downregulation of NF-kB activity [63]. A selenium enriched *Bifidobacterium. longum* DD98 effectively reduced the intestinal and hepatic toxicity of irinotecan (CPT-11), by reducing the pro-inflammatory cytokines IL-1β and IL-18 [64]. Treatment with *Bifidobacterium. breve* NCIMB 702258 decreased proinflammatory cytokines TNF-alpha and IFN-gamma levels [65]. Together, these studies demonstrated that different strains of *bifidobacterium* have the potential to decrease different proinflammatory cytokines and at the same time increase anti-inflammatory cytokines. Summary of the influence of different strains of *bifidobacterium* on inflammatory cytokines is depicted in Table 1.

**Table 1:** *Bifidobacterium* regulates pro-inflammatory and anti-inflammatory cytokines

Strain	Sample	Immunological changes	References
*Bifidobacterium. animalis F1-7*	Lung	↓TNF-α, IL-6, IL-8 ↑IL-10	Lu Y, et al., 2023 [56]
*Bifidobacterium. longum BL-10*	Intestine	↓TLR4 and NF-kB	Dong J, et al., 2022 [57]
*Bifidobacterium. longum BL-10*	Intestine	↓IFN-γ, IL-2, IL-6, IL-17, IL-22, and IL-12	Dong J, et al., 2022 [57]
*Bifidobacterium. Breve* BR03 and B632	Blood	↓TNF-α	Klemenak M, et al., 2008 [58]
*Bifidobacterium adolescents*	Colon	↑IL-10, IL-4 and IL-5	Fan L, et al., 2021 [60]
*Bifidobacterium adolescents*	Colon	↓TNFα, IL-6, IL- 1β, IL-18, IL-22, and IL-9	Fan L, et al., 2021 [60]
*Bifidobacterium. longum BB536 and Bifidobacterium. breve M-16V*	Colon	↓IL-8, MCP-1, and IL-6	Tomosada, et al. [62]
*Bifidobacterium animalis, subsp. lactis strain BB12*	Caco-2 cells	↓IL-8, NF-kB	Wang Z, et al., 2011 [59]
*Se-Bifidobacterium. longum DD98*	Small intestinal epithelial cell	↓IL-1β and IL-18	Zhu H, et al., 2021 [64]

### *Bifidobacterium* enhances anti-tumor action of immunotherapy

Although the immune modulating *bifidobacterium* strains that directly attack cancers are very limited, there are several evidences available on the combination of immunotherapy and *bifidobacterium’s* action in enhancing the anti-cancer potential of immunotherapy. Immune checkpoint inhibitors act on immune checkpoints such as CTLA-4 and PD-1 to enhance the T cell response towards cancer cells. Recent research observed that immune checkpoint inhibitors anti-tumor action is significantly influenced by gut probiotics [34]. Mager et al. reported that *Bifidobacterium. pseudolongum* could not exhibit anti-tumor immunity against colon cancer, bladder cancer and melanoma, whereas its combination with anti-CTLA-4 treatment significantly decreases tumor growth compared to anti-CTLA-4 treatment alone by increasing IFN-γ production mediated via higher spleen TH1 cell activation [66]. Furthermore, this study found that the concentration of a microbial metabolite inosine was significantly higher in *Bifidobacterium. pseudolongum* monocolonized colorectal cancer mice model compared with *colidextribacter* species monocolonized mice [66]. The authors stated that the enhancement of anti-tumor action of CTLA-4 antibody by *Bifidobacterium. pseudolongum* was mediated by inosine-A2AR signaling in T cells [66]. A milestone study by Sivan et. al. showed that *bifidobacterium spp*. enhanced the anticancer action of PD-L1 against melanoma [67]. Another study showed that *Bifidobacterium. bifidum* significantly increased the anti-tumor immunity by enhancing the anti-cancer action of PD-L1 treatment in a melanoma mouse model [68]. A study by Lee et al. observed that *Bifidobacterium. bifidum* treatment together with PD-1 antibody significantly inhibited the tumor growth compared to the monotherapy of PD-1 antibody [27]. *Bifidobacterium. longum* RAPO enhances the anti-tumor immune response of anti-PD-1 therapy in triple negative breast cancer [69].

A very recent study found that *Bifidobacterium*-derived extracellular vesicles (Bif.BEVs) enhanced anti-cancer action of anti-PD-1 therapy in NSCLC. Both *in vivo* and *in vitro* study found that lung cancer cells uptake Bif.BEVs through dynamin-dependent endocytosis pathway. Further, this study demonstrated that Bif.BEVs treatment significantly increased PD-L1 expression in lung cancer cell lines through TLR4-NF-κB pathway [70]. A recent study in liver cancer observed that the combination of *bifidobacterium* or isobutyrate with anti-PD-1 significantly reduced the tumor size. Further, this study demonstrated that the abundance of *bifidobacterium* or levels of isobutyrate in the gut microbiota may assist as predictive markers for immunotherapy response in liver cancer patients [71].

### Carcinogen detoxification by *bifidobacterium*

**Figure 1:**
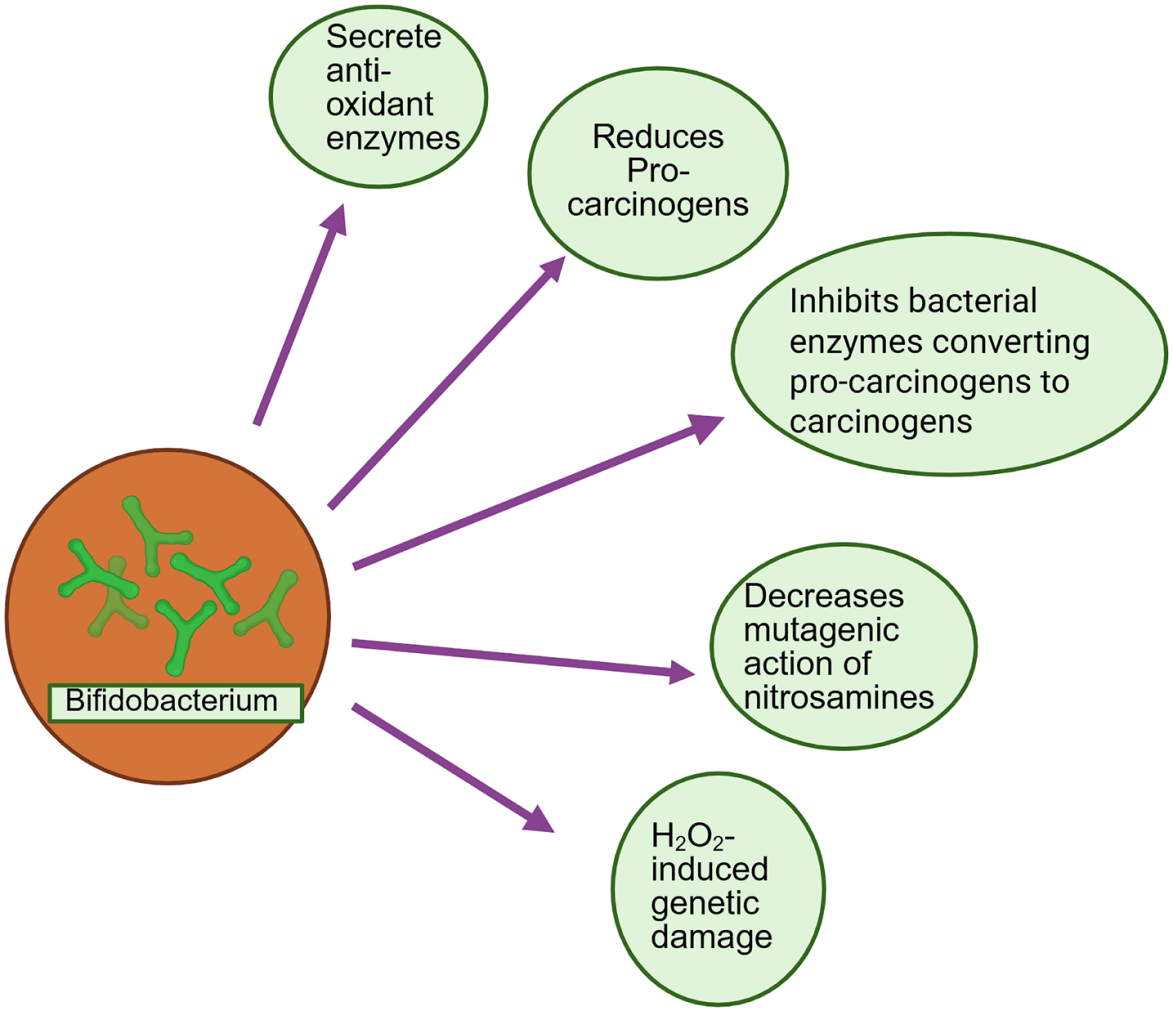
Schematic illustration of carcinogen detoxification by *bifidobacterium*.

Available reports suggested that *bifidobacterium* decreases the production of carcinogens by regulating metabolism in the intestinal flora [72]. *Bifidobacterium* intake decreased the levels of different bacterial enzymes such as nitroreductase, β-glucuronidase, and azoreductase [73, 74]. These bacterial enzymes are responsible for the conversion of procarcinogens to carcinogens [75]. Anti-carcinogenic effects of *bifidobacterium* may also be due to the elimination of procarcinogens from the intestinal flora [1]. Common carcinogenic compounds that exist in food and the atmosphere are polycyclic aromatic hydrocarbons (PAHs), heterocyclic amines (HCAs) pthalic acid esters (PAEs), and mycotoxins [76]. It has been reported that HCAs were bound with *Bifidobacterium. pseudocatenulatum* and *Bifidobacterium. longum* [77]. This may lead to decreases in the absorption of HCA in the intestine and consequently increase the elimination via feces [77]. *Bifidobacterium* can decrease the mutagenic action of nitrosamines. It has been reported that *bifidobacteria* can physically bind to nitrosamines such as HCAs [77]. *Bifidobacterium. animalis* var. lactis Bb12 inhibited the formation of aberrant crypt foci (ACF) by dimethylhydrazine (DMH) in mice. This indicates *bifidobacterium* protected from carcinogen-induced colonic preneoplastic lesions in mice [78]. Another study found that H_2_O_2_-induced DNA strand breaks and overall genetic damage were protected in colorectal cancer cells (HT29clone19A) by human fecal water, which is collected from probiotics (*Lactobacillus acidophilus* 145 and *Bifidobacterium. longum* 913) treated healthy human volunteers [79]. Figure 1 represents the schematic illustration of carcinogen detoxification by *bifidobacterium*.

A study analyzed the effects of dietary probiotic bacteria *Bifidobacterium. lactis (B. lactis)*, resistant starch (RS), and their interaction on colorectal cancer apoptosis. This study revealed that a combination of dietary RS and *Bifidobacterium. lactis* significantly enhanced the cancer cell’s apoptotic response to a carcinogen in the colorectal cancer animal model [80]. *Bifidobacterium* strains were found to decrease cancer cell growth by exhibiting anti-proliferative, pro-apoptotic, and antioxidant attributes. *Bifidobacteria* was shown to accomplish pro-apoptotic action by increasing pro-caspases and Bax proteins and downregulating the anti-apoptotic Bcl-2 proteins [81].

### *Bifidobacterium* regulates cancer cell apoptosis and signaling

Different reports suggest that *bifidobacterium spp*. can regulate the genes involved in cell proliferation and apoptosis [81]. Treatment of a cocktail containing five strains of *bifidobacterium* induced cell death in colon adenocarcinoma cells [82]. Further, *bifidobacterium* treatment decreased the expression of PTGS-2 (20 folds), HER-2 (6.7 folds), and EGFR (4.4 folds) [21]. Another study suggested that aqueous extracts from *Bifidobacterium. bifidum* and *Bifidobacterium. lactis* significantly decreased the proliferation of NSCL cancer cell lines (A549 and H1299). Further, *bifidobacterium* increased apoptosis, enhanced the levels of cleaved poly ADP-ribose polymerase (PARP) and caspase 3. *Bifidobacterium* also decreased expression of MMP-9, which led to decreased invasiveness of lung cancer cells [25]. A recent study suggested that *Bifidobacterium. pseudolongum* reduced non-alcoholic fatty liver disease-associated hepatocellular carcinoma (NAFLD-HCC) in an animal model. *Bifidobacterium. pseudolongum* significantly reduced cell growth via the reduction in the G1/S transition of cell cycle. Furthermore, this study found that *Bifidobacterium. pseudolongum* reduced IL-6/JAK1/STAT3 signaling pathway by the activation of G coupled-protein receptor 43 [83]. *Bifidobacterium. bifidum* reduced tumor growth in the gastric cancer xenograft model. This study further demonstrated that the decreased tumor growth is due to decreased Akt phosphorylation, increased expression of tumor suppressor p53, and apoptotic regulators Bax and Bak proteins. Higher apoptosis in gastric tumor was confirmed by higher expression of cleaved caspase-3 and 9, and PARP [84]. A study provided evidence on the anti-cancer action of exopolysaccharides derived from *Bifidobacterium. breve lw01*. The authors identified that genes responsible for the exopolysaccharide’s biosynthesis are present in this bacterium’s genome as cluster regions with 14 predicted genes. The exopolysaccharides identified in *Bifidobacterium. breve* lw01 are rhamnose, arabinose, galactose, glucose, and mannose. Experiments using head and neck squamous cancer cell lines identified that exopolysaccharides inhibited cancer cell proliferation in a concentration dependent manner by powerfully impacting cell cycle arrest and apoptosis regulating machinery [54]. Exopolysaccharides derived from Bifidobacteria enhance the production of cytokines such as TNF-α and IL-12, thereby promoting T cell-mediated apoptosis in colon cancer cells [85]. Summary of different apoptotic signaling molecules regulated by different strains of *bifidobacterium* are illustrated in Table 2.

**Table 2:** *Bifidobacterium* regulates cancer cell apoptotic signaling molecules

Strain	Cancer types	Apoptotic signaling	References
*Bifidobacterium*	Colon adenocarcinoma cells	↓PTGS-2, HER-2 and EGFR	Asadollahi P, et al., 2020 [21]
*Bifidobacterium. bifidum and Bifidobacterium. lactis*	Non-Small Cell Lung Cancer Cell lines	↑PARP and caspase 3	An J, et al., 2020 [25]
*Bifidobacterium. pseudolongum*	Hepatocellular carcinoma	↓IL-6/JAK1/STAT3 ↑GPCR 43	Song Q, et al., 2023 [83]
*Bifidobacterium. bifidum*	Gastric cancer xenograft model	↓Akt Phosphorylation ↑p53 Bax and Bak, ↑PARP and caspase 3 and 9	Kim S, et al., 2022 [84]
*Bifidobacterium. breve lw01*	Head and neck squamous cancer cell lines	↑Exopolysaccharides	Wang L, et al., 2019 [54]

Moreover, the metabolites produced by *bifidobacterium* exhibit anti-cancer action. Supplementation of *bifidobacterium* via probiotics or prebiotics may increase the levels of blood omega-3 PUFAs. *B. breve NCIM*B 702258 and linoleic acid-supplemented diet enhanced the omega-3 (n-3) fatty acids, such as, eicosapentaenoic acid and docosahexaenoic acid levels in mice adipose tissue [65]. Studies suggested that PUFAs can induce anti-carcinogenesis action [86]. Furthermore, *bifidobacteria* produces short chain fatty acids including acetate, butyrate, and propionate [87, 88]. These metabolites produce anti-cancer action against colon cancer [89, 90]. Butyrate is an important metabolite that exhibits both immunomodulatory and anti-inflammatory properties. Furthermore, butyrate acts as a tumor suppressor which induces anti-tumor action in various cancers [91–93].

### *Bifidobacterium* influences biotransformation of dietary components

Anticancer action of *bifidobacteria* may also be due to the biotransformation of dietary components into anti-cancer molecules. A study using a leukemic mice model showed that dietary pectic oligosaccharides (POS) enhanced the growth of *bifidobacteria* compared to inulin containing diet. It was also shown that (POS) increased the *bifidobacterium* species and increased the concentration of short-chain fatty acids (SCFA) such as acetate, propionate, and butyrate [13]. A study analyzed the effect of two types of rye-bran fractions on the population of *bifidobacrterium*/enterolactone production in intestinal neoplasia animal models. This study demonstrated that rye-bran diet increased the *bifidobacterium* population, whereas the non-fiber diet significantly lowered intestinal *bifidobacterium* level [14]. In addition, the same study found that higher levels of intestinal *bifidobacterium* might be associated with more enterolactone production [14], which likely provides an anti-tumor safeguard against intestinal neoplasia in mice [9]. Several other studies indicated that intake of inulin, a prebiotic fiber increased gut *bifidobacterium* [94]. A human study showed that higher production of *bifidobacteria* through the supplementation of oligofructose and inulin-containing diet [95], and another human study showed that the growth of most *bifidobacteria* was higher with inulin and oligofructose compared to glucose [96]. A recent randomized controlled trial using type 2 diabetes patients showed that, compared to maltodextrin, six weeks of supplementation of a 50/50 mixture of inulin and oligofructose increased fecal *bifidobacteria* and short-chain fatty acids, acetic acid, and propionic acid [97]. Butyrate has been shown as an effective anti-cancer agent by reducing the level of inflammatory cytokines, and act as an inhibitor to histone deacetylase [98].

It has been established by various studies that fructose oligosaccharides and inulin are effective prebiotics. Short-chain fructo-oligosaccharides consist of polysaccharides containing glucose connected to fructose units (Gfn; *n* = </= 4). The oligosaccharides are fermented only in the colon and enhance the *bifidobacteria* growth. The optimal level of fructo-oligosaccharides required for healthy human volunteers to increase fecal *bifidobacteria* is 10 g/d. These non-digestible dietary components specifically enhance the growth of certain gut microbiota such as *bifidobacteria* [99]. One other report suggested that intake of 12.5 g/d dietary fructo-oligosaccharides increased colonic bifidobacteria in human volunteers [100]. Fructo-oligosaccharides (FOS) consist of fructose connected by β-(2→1)-glycosidic bonds with a glucose unit. FOS and inulin are present in many dietary sources such as asparagus, onions, garlic, Jerusalem artichokes, and leeks [101]. Inulin, comprise of fructose moieties connected through (2-1)-d-frutosyl fructose bonds, which is mainly isolated from chicory roots [102]. *Bifidobacteria spp*. can catabolize different kinds of mono-and oligosaccharides. *Bifidobacterium* can ferment dietary fructo-oligosaccharides and inulin [101, 103]. *Bifidobacterium. adolescentis* and *B. thermophilum* fermented both FOS and inulin, Whereas *Bifidobacterium. infantis* and *Bifidobacterium. bifidum* fermented only FOS not inulin. β-fructofuranosidase is the common enzyme found in most of the *bifidobacterium* strains, however, strains that ferment inulin have more hydrolytic activity against fructans. Various gut microbiota showed differences in fermenting carbohydrates by using different pathways. *Bifidobacteria* in particular use a pathway named “bifid shunt” to degrade hexose sugar. The key enzyme in this pathway is fructose-6-phosphoketolase (EC 4.1.2) [104].

Polyunsaturated fatty acids (PUFA) have a direct impact on the level of different gut microbiota. Similarly, gut microbiota influences the bioavailability, and biotransformation of PUFAs [104]. Different studies suggest that omega-3 PUFAs regulate gut microbiota population, increasing the growth of *bifidobacteria* [104–106] and decreasing the growth of *Enterobacteria* [105]. Correspondingly, there is a correlation that exists between probiotic supplementation and fatty acid levels in serum and tissues. Omega-3 polyunsaturated fatty acids (PUFAs) are essential nutrients that display various health benefits. *Bifidobacterium* has been shown to have a positive association with the level of different PUFAs such as the level of DHA and omega-3 PUFAs in breast cancer survivors [107]. Summary of the different dietary constituents which influence the intestinal/colon/fecal *bifidobacterium* population is described in Table 3.

**Table 3:** Dietary components influence intestine/colon/fecal *bifidobacterium* levels

Location	Dietary components	References
*Intestinal Bifidobacterium. dorei/Bifidobacterium.vulgatus*	Pectin-derived oligosaccharides metabolite-Short Chain fatty acids	Bindels LB, et al., 2015, [13]
*Intestinal Bifidobacterium*	Rye-supplemented diets (metabolite -Enterolactone)	Oikarinen S, et al., 2003 [14]
*Colon Bifidobacterium*	Inulin/Oligofructose	Gibson, GR, et al., 1995 [95]
*Intestinal Bifidobacterium*	Inulin/Oligofructose	Rao AV, 1999 [95]
*Fecal Bifidobacterium*	Short-chain fructo-oligosaccharides	Bouhnik, Y et al., 1999 [12]
*Colonic Bifidobacterium*	Fructo-oligosaccharides	Bouhnik Y, et al., 1996 [100]
*Gut Bifidobacterium*	Omega-3 PUFAs	Fu Y, et al., 2021 [105]

*Bifidobacteria* may also metabolize certain drugs into active anti-cancer molecules [108]. Gut microbiota, such as r*uminococcus spp., bacteroides spp*., and *bifidobacterium spp*. are involved in the metabolism of ginseng to ginsenoside Rb1, which has potent anti-cancer action [109]. Another study reported that *bifidobacterium sp*. metabolized ginsenoside Rb2 to 20-O-beta-D-glucopyranosyl-20(S)-protopanaxadiol (compound K) [110]. Anticancer action of compound K has been observed in different cancers such as acute myeloid leukemia, liver, lung, and nasopharyngeal carcinoma [111]. Lapachol (1,4-naphthoquinone) is naturally present in the bark of the lapacho tree. *Bifidobacterium sp*. and *Lactobacillus acidophilus* metabolized lapachol to dehydro-α-lapachone, which showed higher anticancer activity compared to lapachol [112]. Antitumor bioactive molecules formed in soymilk during fermentation by *Streptococcus thermophilus* 14085 and *Bifidobacterium. infantis* 14603 displayed higher anti-tumor activity against colorectal cell lines compared to normal soymilk [113].

## SUMMARY AND FUTURE PERSPECTIVE

**Figure 2:**
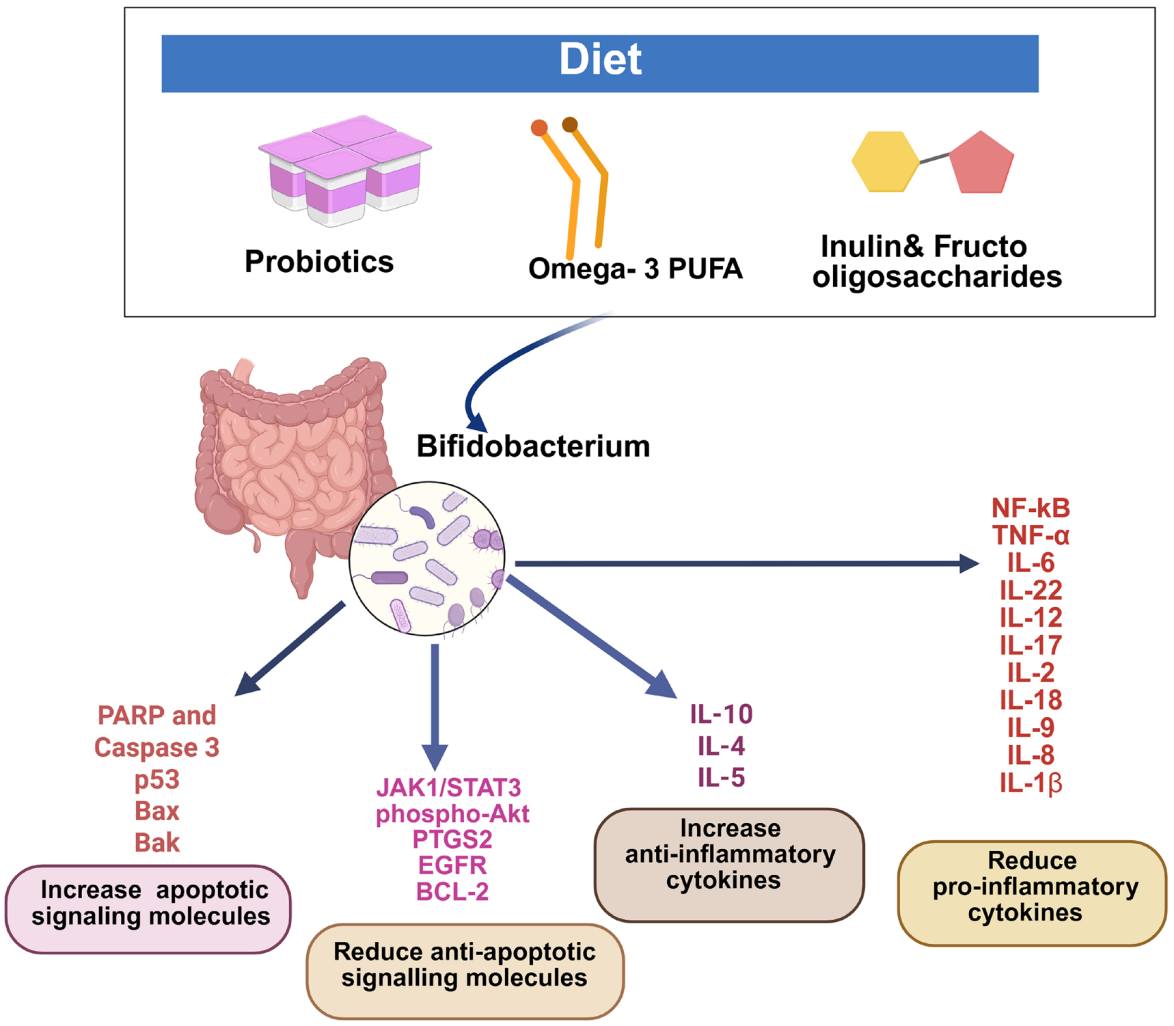
*Bifidobacterium* regulates inflammatory cytokines and apoptotic signals.

Different health benefits of *bifidobacterium* have been identified, such as anti-cancer, immune regulation, and anti-inflammatory effects (Figure 2). Anti-cancer action of *bifidobacterium* has been documented in different tumors and cancer cell types. *Bifidobacterium* species reduced the growth and metastasis of NSCLC [23, 25]. Presence of *Bifidobacterium. breve* in gut microbiota extended the median progression-free survival of NSCLC patients. Different studies reported the levels of different species of *bifidobacterium* provide a prediction on the efficacy and adverse effects of immune checkpoint inhibitor therapy for NSCLC patients [24]. Premenopausal breast cancer patients displayed lower levels of *bifidobacterium spp*. compared to postmenopausal patients [33] yielding to a possibility that should be investigated. TNBC occurs mostly in premenopausal women, and there are few reports suggesting the anti-cancer action of *bifidobacterium* against TNBC [35–38]. Additionally, studies demonstrated that *Bifidobacterium. infantis* increased the anticancer action of doxorubicin for TNBC [35], which should be extrapolated whether this type of combination that can be used to synergize the action of chemotherapeutics yielding to the possibility of minimizing the side effects of the drug that are incurred by increasing the dose of the drug. TNBC is one of the hard-to-treat cancer compared to other types of breast cancers. Chemotherapy is one of the major treatment options for TNBC, and majority of patients develop resistance, and it leads to tumor metastasis. Hence it will be worthwhile to explore the efficacy of *bifidobacterium* in supporting the chemotherapy action in TNBC patients.

The pro-apoptotic action of *bifidobacterium* was established in colon cancer cell lines and in animal models [40–42]. *Bifidobacterium* reduced the level of different colorectal cancer, oncogenic signaling molecules such as EGFR, COX-2, miR-21a and miR-155 [21], and simultaneously enhancing the tumor suppressor miR-145 and miR-15a [45]. A fascinating mechanism of anti-cancer action of *bifidobacterium* is mediated through the immune regulation primarily targeting proinflammatory cytokines [57, 60, 64]. Available studies have demonstrated that the presence of *bifidobacterium* considerably decreased pro-inflammatory cytokines such as TNF- α and IL-6, IL-18, and IL- 1β, whereas the anti-inflammatory cytokines such as IL-10, IL-4, and IL-5 increased appreciably (Figure 2). Most of these changes are detected in intestinal and/or/colonic tissues. Moreover, studies available in various cancer models are limited; therefore, results of these studies should be interpreted with caution. Hence, further studies are required to evaluate the effect of *bifidobacterium* species on specific immune regulators in different types of cancer. Different stains of *bifidobacterium* decrease the levels of different bacterial enzymes, which are responsible for the conversion of dietary procarcinogens to carcinogens [73, 75]. It has also been suggested that different signaling molecules involved in cancer cell proliferation decrease [25, 81]. One of the other mechanisms is the biotransformation of different dietary components into anti-cancer molecules. Various studies demonstrated *bifidobacterium* growth is influenced by specific diets such as inulin, oligofructose [94–96], and PUFA [105–108] (Figure 2). Initiating studies on cancer prevention through a high fiber diet mediated by enriched gut *bifidobacterium* is crucial.

With context to complement the benefit of *bifidobacterium* in enhancing the action of immunotherapy, radiation therapy and chemotherapy, more studies are needed to identify anti-cancer specificity of different *bifidobacterium* species. Recent studies suggest that *bifidobacterium* increases the anti-cancer action of immune check point inhibitors (anti-PD1 and anti-CTLA4) for various cancers [66–71]. It is well documented that chemotherapy and radiation treatment decrease the intestinal microflora including *bifidobacterium* [113–119] and increases pathogenic family members of *enterobacteriaceae* [120, 121]. When designing *in vivo* studies related to the combination of *bifidobacterium* and chemotherapy, it’s imperative to evaluate the presence of intestinal *bifidobacterium*.

In summary, the presence of *bifidobacterium* in the intestinal flora can be heavily influenced by the intake of probiotics as well as a diet rich in fibers like inulin, pectin, oligofructose, and other components such as PUFA. *Bifidobacterium’s* anticancer effects are multifaceted, involving the modulation of immune responses through the regulation of both pro-inflammatory and anti-inflammatory cytokines, influencing apoptotic pathways, and neutralizing carcinogens introduced into the body via diet and miscellaneous other environmental exposures. Consequently, *bifidobacterium* has the potential to complement conventional chemotherapeutic regimens, immunotherapy, and radiotherapy. Nonetheless, further research is necessary to fully understand the specific roles of individual *bifidobacterium* strains and their impact on different types of cancer, particularly in terms of cancer prevention, enhancing standard treatments and reducing tumor proliferation and metastasis.
